# Evaluation of the retinal and choroidal microvasculature in retinitis pigmentosa using OCT-angiography: a meta-analysis

**DOI:** 10.1177/25158414261471361

**Published:** 2026-09-01

**Authors:** Evita Evangelia Christou, Amandeep S. Josan, Robert E. MacLaren

**Affiliations:** Oxford Eye Hospital, Oxford University Hospitals NHS Foundation Trust, Oxford, UK; Nuffield Laboratory of Ophthalmology, Nuffield Department of Clinical Neurosciences, University of Oxford, Oxford OX3 9DU, England, UK; Oxford Eye Hospital, Oxford University Hospitals NHS Foundation Trust, Oxford, UK; Oxford Eye Hospital, Oxford University Hospitals NHS Foundation Trust, Oxford, UK; Nuffield Laboratory of Ophthalmology, Nuffield Department of Clinical Neurosciences, University of Oxford, Oxford, UK

**Keywords:** foveal avascular zone, microcirculation, optical coherence tomography angiography, retinitis pigmentosa, vessel density

## Abstract

**Background:**

Retinitis pigmentosa (RP) comprises a group of genetically and clinically heterogeneous retinal dystrophies characterized by progressive retinal degeneration, leading to deterioration of vision. Optical coherence tomography angiography (OCTA) enables non-invasive assessment of retinal and choroidal microvasculature and has increasingly been used in the assessment of microvascular changes in PR.

**Objectives:**

To assess current evidence regarding the retinal and choroidal microvasculature using optical coherence tomography angiography in patients with RP.

**Design:**

Meta-analysis.

**Data sources and methods:**

A comprehensive literature search was performed in the PubMed database, including studies published up to July 2025. Eligible journal articles were quality and consistency assessed prior to inclusion. Mean difference (MD) and variances were calculated from reported means and standard deviations in patient and control groups. Heterogeneity was evaluated based on the values of *I*^2^ and *p*-values. Group mean differences were reported with 95% confidence intervals (CIs) and 95% predicted intervals (PIs).

**Results:**

Nineteen eligible studies were included for this meta-analysis. The foveal perfusion density of RP patients was significantly lower than that of the control group for superficial capillary plexus, MD = −6.09% (95% CI: −9.75, −2.43), and deep capillary plexus, MD = −4.57% (95% CI: −7.22, −1.91). The parafoveal perfusion density of RP patients was significantly lower than that of the control group for superficial capillary plexus, MD = −8.72% (95% CI: −11.71, −5.74) and deep capillary plexus, MD = −6.55% (95% CI: −12.14, −0.96). The foveal avascular zone area of RP patients was significantly increased as compared to that of the control group for superficial capillary plexus, MD = 0.10 mm^2^ (95% CI: 0.02, 0.17), and deep capillary plexus, MD = 0.12 mm^2^ (95% CI: 0.02, 0.23). The perfusion density in the choriocapillaris of RP patients was not significantly lower than that of the control group in the foveal region, MD = −1.41% (95% CI: −6.73, 3.91), but was significantly lower in the parafoveal region, MD = −2.44% (95% CI: −3.85, −1.03). Significant heterogeneity was present across studies, resulting in PIs encompassing zero difference for all measures.

**Conclusion:**

The findings suggest an association between retinitis pigmentosa and reduced retinal and choroidal microvasculature parameters. However, substantial interstudy heterogeneity and wide PIs limit the certainty and generalizability of the results. Microvascular attenuation is likely occurring secondary to retinal degeneration and may share a similar mechanism to the peripheral large vessel attenuation, which is typically described peripherally in advanced RP.

## Introduction

Retinitis pigmentosa (RP) comprises a group of genetically and clinically heterogeneous inherited retinal dystrophies that are characterized by progressive degeneration of photoreceptors, leading to deterioration of vision.^1^ Global prevalence is estimated at approximately 1 in 4000, with over 1 million affected individuals worldwide.^2^ To date, RP has been associated with over 50 causative gene mutations with variable inheritance patterns such as autosomal dominant, autosomal recessive, or X-linked. There is a complex underlying pathophysiology with several factors implicated in the onset and progression of the disorder. Typically, RP affects the outer retinal layers. It is considered a primary degeneration of rod photoreceptors followed by secondary cone involvement, in turn causing subretinal microinflammation and retinal pigment epithelium hyperplasia resulting in the classic pigmentary retinopathy phenotype. Furthermore, remodeling of inner retinal layers with ganglion cell loss has been reported.^3,4^

Recently, there is increasing evidence that apart from photoreceptor degeneration, microvascular changes in retinal layers and the choriocapillaris (e.g., perivascular cuffing, arteriolar attenuation, reduced ocular blood flow) have been identified as a co-existing factor with the progression of the disorder. These vascular alterations have also been hypothesized to be part of the pathogenic process of RP and reported in histopathologic and experimental studies.^5,6^ The advent of OCT-angiography (OCTA) has offered the opportunity to assess noninvasively the retinal and choroidal microvasculature changes that could serve as potential surrogate biomarkers for various disorders, including RP. Several studies have indicated the presence of microvasculature alterations in patients with RP and examined the association of OCTA metrics, such as vascular density and foveal avascular zone, with RP progression; however, their outcomes serve unequivocal perspectives. A thorough understanding of microcirculation integrity in RP remains an unmet need; this could facilitate further progress in the development of appropriate therapies and yield new insights on potentially modifiable causes of progression.^7,8^

Previously, Ling et al.^8^ performed a meta-analysis to assess the retinal and choroidal microcirculation characteristics using OCTA in patients with RP. Their goal was to identify potential diminishment of the vascular component, as this seems to be an element contributing to disease progression. Their quantitative synthesis included seven original studies that were considered eligible for analysis and indicated both retinal and choroidal vascular attenuation in RP eyes when compared to controls.^9–15^ Following their analysis, further studies have been published in an attempt to clarify the potential role of vascular alterations in the pathophysiology and progression of RP.^16–47^ Our aim was to perform a larger-scale analysis including 19 eligible studies to update the existing meta-analysis, verify the validity of the previous findings, and comprehensively quantify further changes with regard to the microvascular component.^16–27^

The aim of this meta-analysis was to evaluate the retinal and choroidal microvasculature as depicted in OCTA in patients with RP. Herein, we examine the current evidence and assess the robustness of the studies’ data pertaining to the insight into the pathogenesis of the disorder and the clinical usefulness of potential OCTA microvasculature biomarkers in RP.

## Methods

### Literature search

This meta-analysis was conducted in accordance with the PRISMA guidelines.^48^ A literature search was performed using the NCBI PubMed/MEDLINE database to identify relevant published articles from inception up to July 2025. The search strategy employed was tailored to the research question; we attempted to discern if there was truly a connection between OCTA parameters and RP. The respective inclusion and exclusion criteria were considered. All articles in English were considered eligible. The following keywords were used as search terms: “retinitis pigmentosa,” “optical coherence tomography angiography,” “microcirculation,” “vessel density,” “foveal avascular zone.” To identify all relevant research articles, the reference list of eligible retrieved studies was carefully reviewed. The current meta-analysis is based on previously published studies and does not include any studies performed by the authors; therefore, informed patient consent and ethical approval were not required.

### Eligibility criteria

To perform the respective meta-analysis, we adhered to the guidelines of “Meta-analysis of Observational Studies in Epidemiology.” Our search algorithm was structured to identify studies that analyze microvasculature as seen in OCTA in patients with RP. The inclusion criteria that studies needed to fulfill to be considered eligible for inclusion in our meta-analysis were (1) original studies: cross-sectional, case–control, or prospective design; (2) RP diagnosis based on established diagnostic criteria; (3) RP patients and healthy controls recruited; (4) sample size of the study of at least 10; (5) data of OCTA measurements reported as mean and standard deviation; (6) studies provided data on retinal and/or choroidal vascular features; (7) outcomes of the studies included foveal and parafoveal perfusions at superficial and deep retinal capillary plexus and choriocapillaris, and foveal avascular zones at superficial and deep retinal capillary plexus. A critical point to consider is that OCTA vascular metrics are reported with variable terms among studies referring to the same parameter; therefore, we included all metrics consistent with the percentage (%) of perfused vasculature area per unit area in the region of measurement. Studies were excluded based on the following criteria: (1) case reports, letters to the editor, conference abstracts, non-English studies, animal/experimental studies, reviews, and meta-analyses; (2) studies with insufficient data; (3) low-quality studies.

### Study quality assessment

Eligible studies were identified using the following selection process, while duplicate and non-relevant articles were discarded. An initial screening of titles and abstracts of the studies yielded from the literature search was performed, followed by an assessment of the full texts to identify possible relevant articles. Studies that did not meet the initial eligibility criteria were not considered further. The methodologic quality of the included studies was assessed and graded for the eligible articles obtained from the literature search to assess their quality. The Cochrane system for assessment of the studies was employed.^49^ Risk of bias among the studies based on the following criteria was evaluated: random sequence generation, allocation concealment, blinding of participants and personnel, blinding of outcome assessment, incomplete outcome data, selective reporting, and other sources of bias. Funnel plots were used to assess the presence of various types of publication bias. Sunset power-enhanced funnel plots using the standard error and the effect sizes were used to demonstrate statistical power in each study included in the meta-analysis. Forest plots of meta-analysis results concerning each parameter were produced, allowing for an overall effect size estimation.

### Data extraction

Information collected from eligible and relevant studies included: first author’s name, publication year, country where the study was conducted, study design, sample size, number of male and female participants, mean age, OCTA equipment type, and relevant OCTA parameters as outcomes.

### OCTA metrics demonstration

In this meta-analysis, we presented the OCTA metrics of vascular densities in the retinal capillary layers and choriocapillaris by using mean differences as the summary statistic. We used the term perfusion density to represent the vascular density metric calculated in percentage (%) among the studies. This refers to the area of perfused vascularity in the area of measurement; however, different terminology may be employed among the studies, and it is more commonly reported as vessel density. To achieve reliable outcomes, we did not include other OCTA parameters of retinal and choroidal microvasculature which were included in some studies, such as vessel density (mm/mm^2^), flow density (mm^2^), fractal dimension, skeleton density and vessel length density. In addition, we analyzed the area of FAZ (mm^2^) in distinct retinal capillary layers, the superficial and the deep plexus.

Statistical analysis: All statistical analyses were performed in R (version 4.5.0)^50^ using the *metafor* package.^51^ Heterogeneity was evaluated using the chi-squared test based on the values of *p* and *I*^2^. Results were reported as mean difference (MD) between patients and controls and estimates provided with 95% confidence intervals (CI) and 95% prediction intervals (PI).

## Results

### Search results

**Figure 1.**
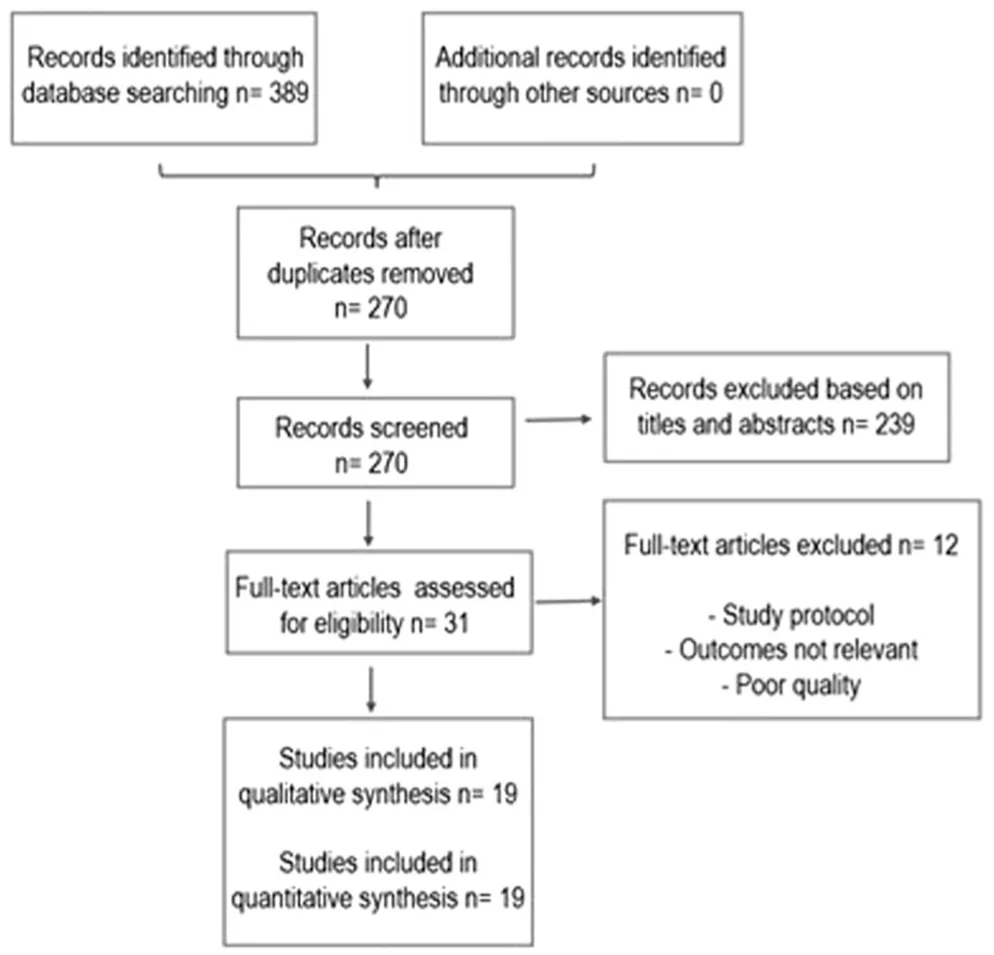
Flow diagram for the article search process for meta-analysis.

Through our literature search, we identified 389 articles, and upon removing 270 non-relevant studies, 31 were selected for the full-text screening. Ultimately, 19 eligible studies were included in this meta-analysis. The flow diagram (Figure 1) indicates the search process for this meta-analysis. Table 1 summarizes the study characteristics and results.

**Table 1.** Summary of study characteristics and results included in the meta-analysis.

Study	Design	Age mean (SD) p/c	Number of eyes (patients) p/c	Gender (F/M) p/c	VA mean (SD) p/c	Macula scan area F, foveal; PAF, parafoveal; PEF; perifoveal (mm^2^)	Study of OCTA characteristics					OCTA device	Quality score
							FAZ		Perfusion macula				
							SCP	DCP	SCP	DCP	CC		
Parodi et al. (2017)^10^	Observational, cross-sectional	53 (18)	32 (16)	6/10	0.50 (0.30)	PAF 3 × 3	+	+	+	+	+	Heidelberg	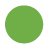
		53 (17)	30 (30)	8/14	0.0 (0.0)								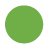
Toto et al. (2016)^11^	n/a	40.1 (7.3)	26 (14)	6/8	0.5 (0.2)	F 1 × 1	−	−	+	+	+	Optovue	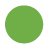
		42.2 (6.5)	24 (24)	10/14	0.0 (0.0)	PAF 2.5 × 2.5							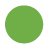
Sugahara et al. (2017)^12^	Prospective, observational	49.9 (17.6)	68 (68)	36/32	0.16 (0.38)	PAF 3 × 3	+	+	+	+	+	Optovue	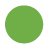
		54.4 (19.9)	32 (32)	20/12	0.11 (0.09)								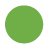
Inooka et al. (2018)^17^	Retrospective, observational	48.3 (17.3)	53 (53)	27/26	0.2 (0.25)	PAF 3 × 3	+	n/a	+	+	−	Zeiss	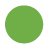
		52.7 (15.4)	46 (46)	21/25	0.0 (0.0)								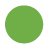
Alnawaiseh et al. (2019)^15^	Prospective	42.4 (14.11)	20 (20)	11/9	0.54 (0. 38)	F 1 × 1	+	+	+	+	+	Optovue	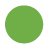
		41.47 (13.54)	21 (21)	12/9	0.0 (0.06)	PAF 2.5 × 2.5							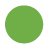
Takagi et al. (2018)^13^	Case-control	46.8 (12.6)	32 (32)	18/14	0.11 (0.07)	PAF 3 × 3	+	+	−	−	−	Optovue	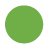
		50.3 (10.0)	14 (14)	11/3	0.08 (0.05)								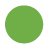
Jauregui et al. (2018)^18^	Retrospective	44.1 (18.45)	28 (28)	11/17	0.28 (0.3)	PAF 3 × 3	+	+	+	+	+	Heidelberg	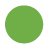
Koyanagi et al. (2018)^9^	Cross-sectional	42.65 (9.17)	73 (73)	36/37	n/a	F 1 × 1	+	+	+	+	−	Optovue	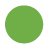
		38.40 (8.50)	36 (36)	20/16		PAF 2.5 × 2.5							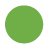
Wang et al. (2019)^14^	Prospective	38.7 (10.5)	40 (20)	9/11	n/a	F 1 × 1	+	−	+	−	−	Zeiss	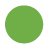
		42.3 (15.7)	26 (13)	8/5									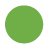
Arrigo et al. (2019)^16^	Observational, cross-sectional	43.2 (12.50)	32	16/16	0.21 (0. 34)	PAF 3 × 3	−	−	+	+	+	Topcon	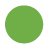
		42.8 (11.2)	32	16/16	0.0 (0.0)								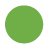
Hagag et al. (2019)^22^	Prospective, cross-sectional	49.6 (22.8)	20 (13)	10/3	0.11 (0.16)	PAF 3 × 3	−	−	+	+	−	Optovue	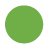
		48.5 (23.7)	34 (26)	11/15	0.04 (0.09)	PEF 6 × 6							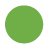
Shen et al. (2020)^19^	Prospective, observational	39.5 (14.03)	34	17/17	0.36 (0.31)	F n/a	−	−	+	+	−	Optovue	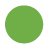
			48			PAF 3 × 3							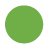
						PEF 6 × 6							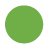
Atta Allah et al. (2020)^20^	Prospective, cross-sectional	26.9 (6.4)	30 (30)	19/11	0.9 (0.5)	F 1 × 1	+	+	+	+	+	Optovue	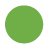
		27.4 (4.9)	24 (24)	16/8	0.05 (0.05)	PAF 2.5 × 2.5							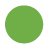
Sabbaghi et al. (2021)^23^	Cross-sectional	40.19 (14.15)	74 (37)	15/22	0.85 (0.82)	F 1 × 1	+	n/a	+	+	−	Optovue	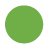
		37.54 (8.82)	100 (50)	28/22	0.05 (0.08)	PAF 3 × 3							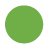
						PEF 6 × 6							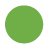
Hong et al. (2021)^24^	Cross-sectional	50.4 (11.5)	30 (17)	7/10	n/a	F 1 × 1	−	−	+	+	−	Optovue	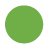
		51 (12)	25 (25)	15/10		PAF 3 × 3							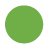
Ong et al. (2021)^25^	Retrospective	43.7 (18.7)	20 (12)	6/6	0.07 (0.1)	F 1 × 1	+	n/a	+	+	+	Optovue	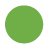
		38.3 (11.3)	19 (10)	8/2	0.04 (0.08)	PAF 3 × 3							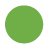
Deutsch et al. (2022)^27^	Retrospective	47.5 (14)	21	13/8	n/a	PAF 3.2 × 3.2	+	+	+	+	+	Heidelberg	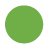
		42.7 (12)	41	27/14									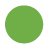
Giansanti et al. (2022)^21^	Observational, retrospective, cross-sectional	50.48 (16.13)	53 (26)	13/13	0.18 (0.25)	PAF 3 × 3	+	−	+	+	+	Nidek	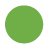
		43.22 (15.71)	38 (19)	13/6	0.0 (0.0)								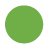
Yilmaz et al. (2023)^26^	Retrospective	35 (5.22)	60 (60)	28/32	0.3 (0.16)	F 1 × 1	+	n/a	+	+	−	Optovue	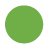
		23 (5.04)	52 (52)	24/28	0.0 (0.0)	PAF 3 × 3							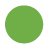
						PEF 6 × 6							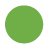

p/c, patients/controls.

### Retinal capillary plexus: Foveal perfusion analysis

**Figure 2.**
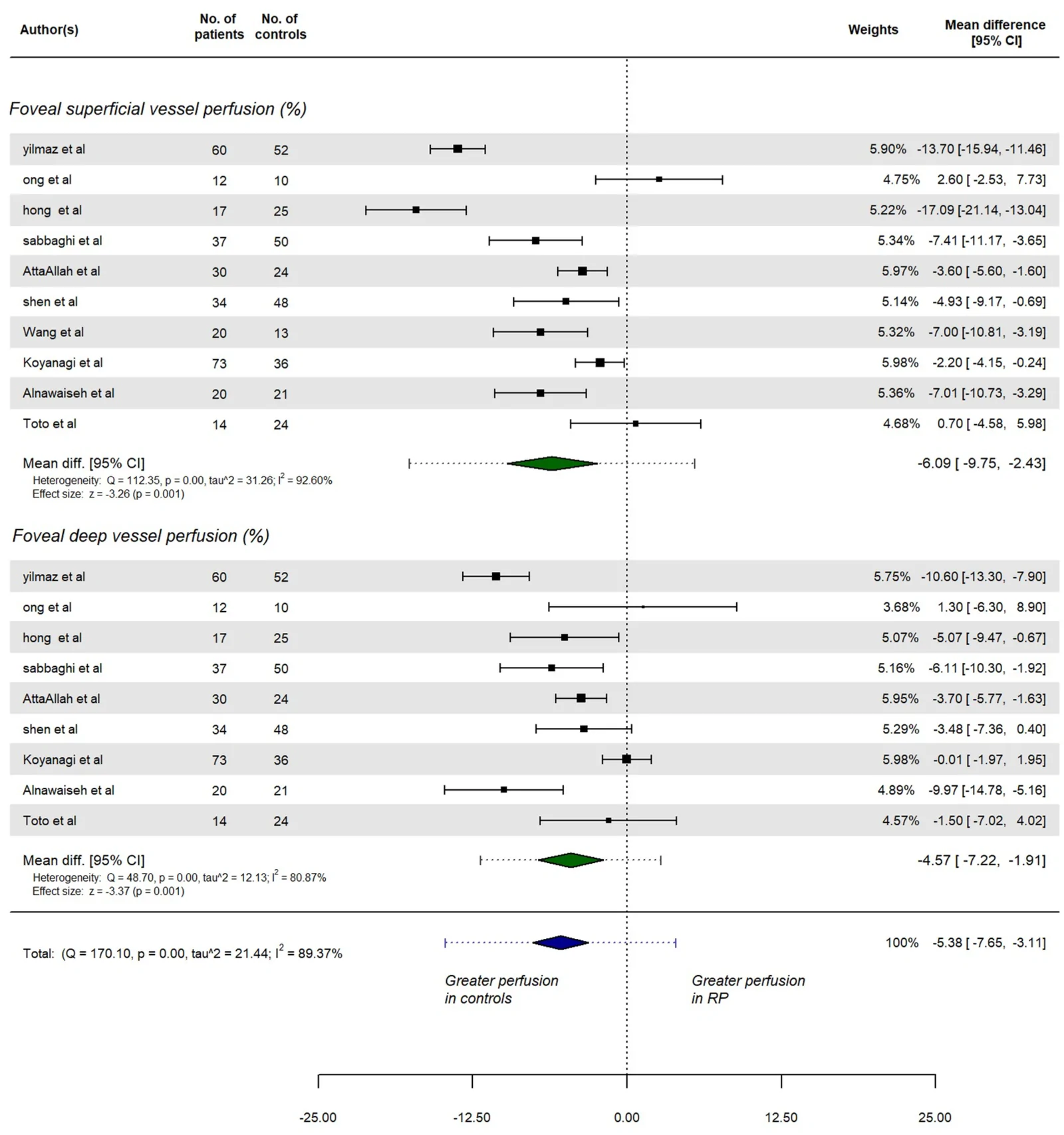
Forest plot with effect size and 95% CIs (solid error bars) shown for both foveal superficial and deep vessel perfusion. The overall mean effect size are shown as the green diamonds in each parameter case and blue diamond for the overall combined effect. The widths of the diamond vertices demonstrate the 95% CIs for the mean and the dotted error bars show the 95% PIs which attempt to predict future values given the effect size and heterogeneity observed. *p*-values for effect sizes are reported based on the CIs, not PIs. CI, confidence interval; PIs, prediction intervals.

Retinal capillary plexus perfusion in the foveal area was evaluated in 10 studies for the superficial and 9 for the deep capillary plexus, including 317 RP patients and 303 controls. The foveal perfusion density values of RP patients in both superficial and deep capillary plexus were significantly lower than those of the control group with a superficial foveal perfusion mean difference of −6.09% (95% CI: −9.75, −2.43), (*p* = 0.001), and a deep foveal perfusion mean difference of −4.57% (95% CI: −7.22, −1.91; *p* = 0.001). Heterogeneity was assessed to be very high among studies, with *I*^2^ = 92.6% and 80.87% for foveal superficial and deep vessel perfusion, respectively, resulting in wide PIs (Figure 2).

### Retinal capillary plexus: Parafoveal perfusion analysis

**Figure 3.**
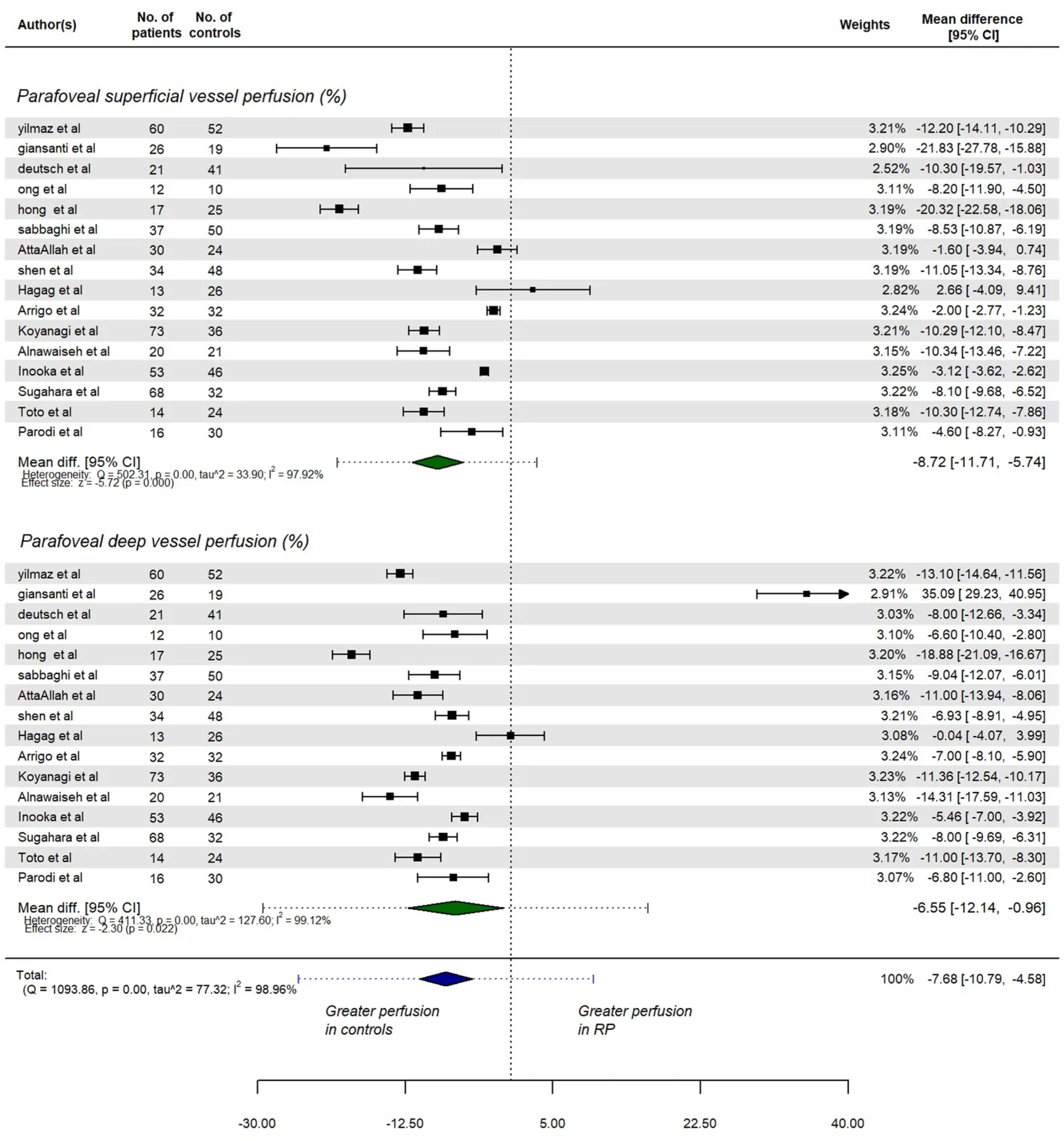
Forest plot with effect size and 95% CIs (solid error bars) shown for both parafoveal superficial and deep vessel perfusion. CI, confidence interval.

Retinal capillary plexus perfusion in the parafoveal area was evaluated in 16 studies for the superficial and 16 for the deep capillary plexus, including 554 RP patients and 516 controls. The parafoveal perfusion density values of RP patients in both superficial and deep capillary plexus were significantly lower than those of the control group, with a superficial parafoveal perfusion mean difference of –8.72% (95% CI: −11.71, −5.74; *p* = 0.000), and a deep parafoveal perfusion mean difference of −6.55% (95% CI: −12.14, −0.96; *p* = 0.022). Heterogeneity was assessed to be very high among studies, with *I*^2^ = 97.92% and 99.12% for parafoveal superficial and deep vessel perfusion, respectively, also resulting in extremely wide PIs (Figure 3).

### Choriocapillaris perfusion analysis

**Figure 4.**
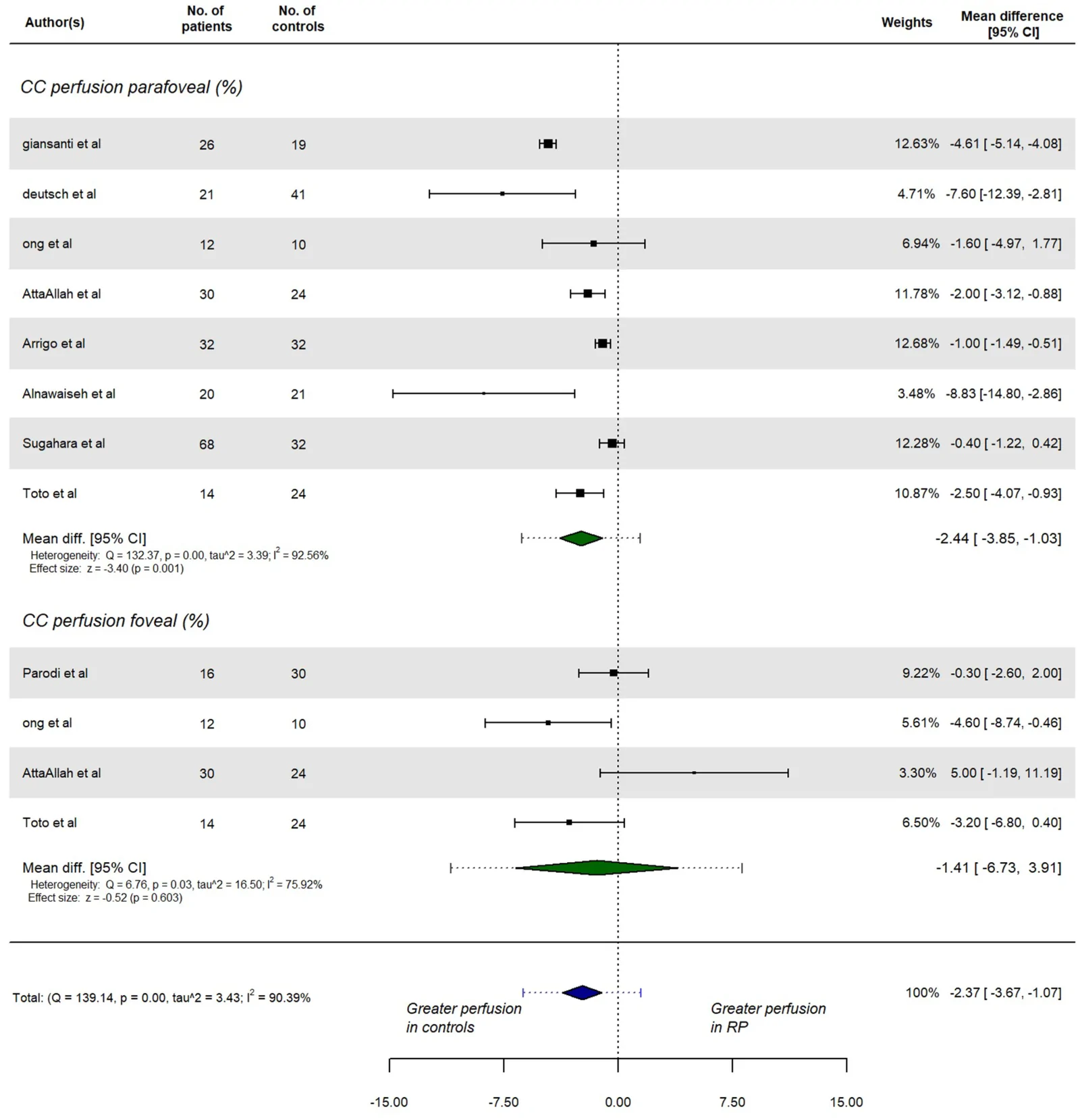
Forest plot with effect size and 95% CIs (solid error bars) shown for both parafoveal and foveal choriocapillaris perfusion. CI, confidence interval.

Choriocapillaris perfusion was evaluated in eight studies for the parafoveal and four studies for the foveal area, including 267 RP patients and 233 controls. The choriocapillaris perfusion density values of RP patients in both the parafoveal and foveal area were lower than those of the control group, with a parafoveal perfusion mean difference of –2.44% (95% CI: −3.85, −1.03) (*p* = 0.001), and a foveal perfusion mean difference of −1.41% (95% CI: −6.73, 3.91; *p* = 0.603). Heterogeneity was assessed to be very high among studies, with *I*^2^ = 90.39% and 92.56% for choriocapillaris perfusion in the parafoveal and foveal area, respectively (Figure 4).

### FAZ analysis

**Figure 5.**
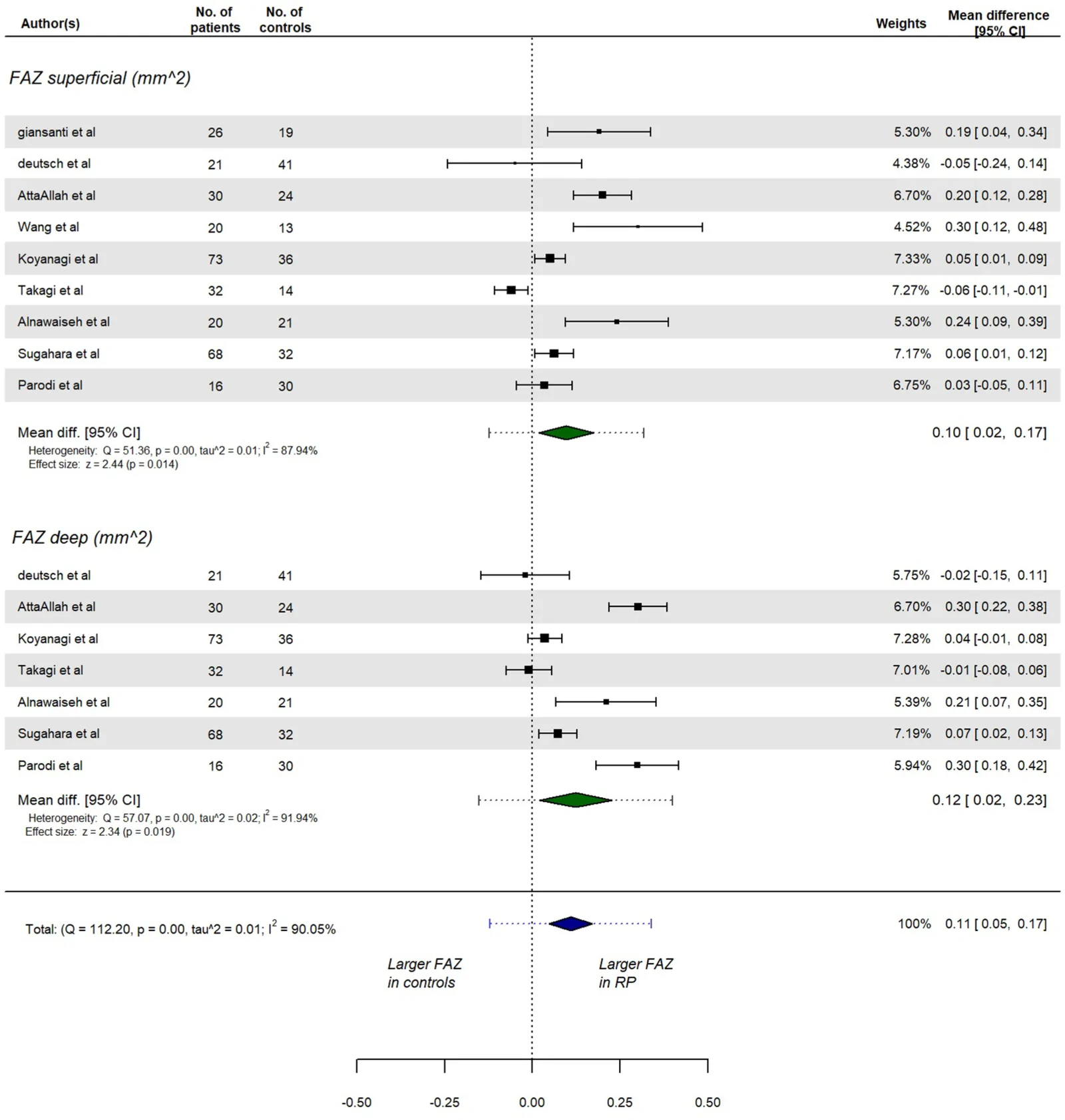
Forest plot with effect size and 95% CIs (solid error bars) shown for both superficial and deep foveal avascular zone. CI, confidence interval.

Foveal avascular zone area was evaluated in nine studies for the superficial and seven for the deep capillary plexus, including 355 RP patients and 230 controls. The foveal avascular zone area of RP patients in both superficial and deep capillary plexus was significantly increased compared to that of the control group, with a superficial FAZ mean difference of 0.10 mm^2^ (95% CI: 0.02, 0.17; *p* = 0.014), and a deep FAZ mean difference of 0.12 mm^2^ (95% CI: 0.02, 0.23; *p* = 0.019). Heterogeneity was assessed to be very high among studies, with *I*^2^ = 87.94% and 91.94% for FAZ area in the superficial and deep capillary plexus, respectively (Figure 5).

The Supplemental section shows funnel plots and sunset power-enhanced funnel plots in order to assess both potential publication bias and the statistical power of each included study.

## Discussion

The pathophysiological mechanism of RP involves progressive degeneration of outer retinal layers, while the exact role of ocular hemodynamics in the context of the disorder has not been fully elucidated.^5,6^ Changes in ocular blood flow have been reported by several studies providing evidence on impaired retinal and choroidal circulation in advanced RP using variable imaging techniques. These findings have been consistent with histopathological changes indicating photoreceptor cells and RPE apoptosis as well as retinal vascular remodeling.^5,6^

This updated meta-analysis aims to reassess potential relationships between retinal and choroidal microvasculature as evaluated by OCTA and the disease course of retinitis pigmentosa. In this study, we identified a statistically significant association in reduction of microvasculature parameters in RP patients when compared to controls, namely diminishment of vascular perfusion and FAZ enlargement. These findings support the prevailing hypothesis that the vascular component is attenuated in RP compared to controls and are in line with the outcomes of an earlier meta-analysis by Ling et al.^8^

Notably, the heterogeneity among the analyzed studies was found to be substantial for all analyzed OCTA metrics, resulting in wide PIs. Confounding factors such as the design of the included studies, the baseline characteristics of the participants, genotype, differing OCTA devices, variable scanning protocols, and the variation of methods of quantitative analysis should be unequivocally considered as contributors to the variation of the studies’ outcomes. Nevertheless, phenotypically, all patients had very similar rod photoreceptor-driven degeneration that led to secondary cone loss via a genetic mechanism, and for this reason, the data are consistent, and much can be interpreted from the observations. The authors suggest that the largest contributor to heterogeneity, however, may be the variation in disease stages present in the patients analyzed across all studies. RP is a progressive disorder, and retinal and choroidal microvasculature alterations are known to evolve with disease severity, ranging from subtle early changes to marked vascular attenuation in advanced stages. The lack of stratification by disease stage across studies therefore limits direct meaningful comparability of reported perfusion metrics and is likely a source of significant heterogeneity. Consideration of a cohort of similarly progressed RP patients would likely only be possible via a new prospective study, making this source of heterogeneity difficult to resolve. In addition to the heterogeneity of disease stage, the genetic heterogeneity of RP represents an additional source of variability. RP encompasses a broad spectrum of inherited retinal dystrophies caused by mutations in numerous genes, each potentially associated with different disease trajectories and phenotypic expressions. These genetic differences may influence the degree and pattern of retinal and choroidal vascular involvement, yet most studies included in this analysis did not stratify results according to genetic subtype. The presence of cystoid macular edema is another factor that may influence OCTA outcomes. Structural distortion of the retinal layers may affect signal quality and floe detection, potentially leading to underestimation or variability in microvasculature parameters. Variability related to OCTA acquisition and analysis represents an additional potential source of heterogeneity in this meta-analysis. Parameters such as device manufacturers, scanning protocols, segmentation, and image processing algorithms can substantially influence quantitative vascular metrics, making them not directly comparable. These heterogeneous OCTA methodologies across studies potentially contribute to the high *I*^2^ values, the wide PIs, and, therefore, uncertainty of the pooled estimates. Differences in scanning protocols and resolution may lead to invalid estimations of vascular density, while segmentation inconsistencies may affect layer-specific measurements. The pooled results should, therefore, be interpreted cautiously as they may partially reflect methodological variability rather than purely biological differences, while standardization of OCTA acquisition protocols and analysis methods will be essential for improving comparability across future studies. Funnel plots (Supplemental Figures 1(a)–4(a)) do not show any indications of publication bias; however, the sunset power-enhanced funnel plots (Supplemental Figures 1(b)–4(b)) suggest that many of the studies are likely to be significantly underpowered to detect microcirculation differences between patient and control groups.

Two studies that were included in this meta-analysis contributed the most to the heterogeneity, as they provide results that are counter to the others. In particular, Giansanti et al.^21^ reported higher perfusion values in the deep capillary plexus in RP patients compared to healthy counterparts. In addition, the study by Tagaki et al.^13^ indicated a significant reduction in FAZ area that is not in line with the other studies reporting FAZ enlargement. These outcomes differ significantly compared to those of the rest of the studies and may be due to a variety of possible reasons including natural variability, study cohort and baseline characteristics, disease staging and progression, and the intrinsic limitations of OCTA technologies. These study results likely have oversized contributions toward the observed high levels of heterogeneity.

The photoreceptor cells and the retinal pigment epithelium are well-established features implicated in the pathogenesis of RP.^2^ The role of choriocapillaris is a crucial parameter since choroidal tissue regulates several aspects of retinal nutrition and metabolism. Even though the exact underlying pathophysiological mechanism remains unclear, previous histopathological analysis has demonstrated loss of choriocapillaris in vivo human eyes. In addition, several studies have indicated diminished choroidal blood flow in patients with RP.^5,6,46^ Our analysis provides supporting evidence for decreased choriocapillaris perfusion in eyes with RP as compared to controls, with significant differences in the parafoveal area, however not reach statistical significance in the foveal region. A contributing factor to this non-significant result may be that very few studies have assessed choriocapillaris perfusion focusing on the foveal area, and of those that have consist of small sample sizes, potentially affecting this outcome.

This meta-analysis has also shown that significant enlargement of FAZ area occurs in both superficial and deep retinal layers in RP. The rarefaction of retinal capillaries and potential vascular insufficiency in the course of the disease could lead to FAZ deformation, providing plausible explanations of the above findings. Apart from measurements in the FAZ area, additional observations on other parameters such as FAZ circularity and perimeter could shed light on more detailed foveal microvasculature alterations in the context of RP.

The underlying mechanism of retinal and choroidal blood flow impairment and the exact cause-effect relationship between ocular hemodynamics and structural changes in RP remain to be fully clarified.^5,6,46^ One hypothesis is that blood flow impairment to surviving retinal regions may occur due to a decrease in oxygen and nutrient needs of the atrophic retina. It has been postulated that outer retinal degeneration may lead to a decrease in metabolic demands and a subsequent vascular remodeling.^46^ A different view argues that retinal atrophic changes may be related to decreased ocular perfusion and retinal vascular attenuation since high blood levels of a potent vasoconstrictor, endothelin-1, have been identified in patients with RP.^46^ Although it was previously suggested that the vascular component may play a primary role in the pathophysiology of the disorder, the argument that the decrease in retinal perfusion is secondary to photoreceptor degeneration has also been proposed.^46^ However, based on the available evidence, it remains unclear whether vascular changes represent a primary pathogenic process or occur secondary to photoreceptor loss. While this latter hypothesis is biologically plausible, a loss of photoreceptors would result in a reduction in metabolic requirements in the subretinal space and potential physiological vasoconstriction in the choroidal circulation; causality cannot be established from the current data.

A pivotal aspect to highlight is the variation in the way that vessel density metrics are assessed among different OCTA equipment and the different terminology used along the studies. Each OCTA modality can utilize variable OCTA metrics to quantify retinal and choroidal microvasculature; we included studies which reported vascular density metrics consistent with the percentage of perfused vasculature area per unit area, measured in percent (%). In this meta-analysis, we used the term perfusion density to represent this vascular metric, which is commonly reported as vessel density.

Although considering exclusively moderate to high quality studies based on the Cochrane system in our meta-analyses, some potential limitations should be inevitably considered. First, several factors may have influenced the pooled estimates of our meta-analyses, such as the methodological heterogeneity among studies with regard to the OCTA metrics, the scanning protocols, the equipment, the selection of participants, and, in particular, the possible diverse severity of the disease within and across each study. Another limitation is the lack of reported genotyping, which may be particularly critical since variable gene mutations may lead to phenotypic variability, including vascular patterns. Moreover, the variability in the severity of the disorder may have resulted in over- or underestimation of the true effect sizes. In addition, some of the included studies evaluated both eyes of the participants, failing to adjust for the inter-eye correlation in their analyses, consequently increasing the likelihood of overestimating the correlation between retinal microvascular measurements and RP. We acknowledge that limiting the search to PubMed and MEDLINE may have resulted in omission of gray literature or conference abstracts; however, this ensures a certain degree of consistency of detail reporting necessary to avoid increasing heterogeneity even further. Finally, we should consider the intrinsic limitations of the OCTA technology, such as differing analysis software in the devices, lack of complete standardization, and potential imaging artifacts that may explain the significant heterogeneity of results across studies. Perfusion density measurements in OCTA may vary depending on scan size and device-specific algorithms. The variability in scanning protocols and OCTA devices using different resolution, segmentation, and thresholding methods across the studies may limit direct comparability of the results and should be considered when interpreting the findings. To improve the reliability of the findings, sub-analysis of foveal and parafoveal regions was performed; however, because of inconsistent reporting across studies, a formal analysis based on device model was not possible. In addition, lack of standardization of OCTA methodology in choriocapillaris assessment may pose challenges in appropriate interpretations. This layer is often manually segmented or derived using different device-specific software and algorithms, resulting in variability across studies. Also, not all OCTA systems provide a standardized choriocapillaris perfusion metric, and measurements may be reported either as area (mm^2^) or percentage (%). Although the studies included in the meta-analysis reported perfusion density in percentage, the methods used to derive this metric varied, representing a limitation of the present analysis. Sub-analysis of foveal and parafoveal choriocapillaris regions was performed to improve reliability. Artifacts may interfere with precise evaluation of the microvasculature. Projection artifacts may affect visualization of retinal and choroidal vessels, while this restriction mainly pertains to the imaging of the deep layers. Motion artifacts are associated with the inability to maintain fixation stability in patients with RP due to long disease duration and severe visual impairment.

In conclusion, this meta-analysis suggests an association between retinal and choroidal microvasculature impairment and RP, indicating reduced vascular density compared with controls. However, substantial interstudy heterogeneity and variability in OCTA methodologies may limit the certainty and generalizability of these results and should be considered when interpreting these findings. In the presence of large heterogeneity, it is recommended that 95% PIs are calculated in addition to only 95% CIs as reported by Ling et al.^8^ PIs provide more realistic error bars based on available evidence and interstudy variability and minimize type 1 errors. Longitudinal comprehensive studies from the early stages of the disease could provide further evidence for the sequelae of the events in the course of RP, such as outer retinal layer degeneration and vascular attenuation. Characterization of genetic mutations and standardized staging could eliminate dilemmas in our understanding of the underlying mechanisms. Assessment of hemodynamic structure in OCTA is a promising area of research that could elucidate the pathogenesis, provide guidance for future directions, and serve as an exploratory outcome measure in clinical trials assessing novel therapies.

## Supplemental Material

sj-docx-1-oed-10.1177_25158414261471361 – Supplemental material for Evaluation of the retinal and choroidal microvasculature in retinitis pigmentosa using OCT-angiography: a meta-analysis Supplemental material, sj-docx-1-oed-10.1177_25158414261471361 for Evaluation of the retinal and choroidal microvasculature in retinitis pigmentosa using OCT-angiography: a meta-analysis by Evita Evangelia Christou, Amandeep S. Josan and Robert E. MacLaren in Therapeutic Advances in Ophthalmology

## Declarations

**Ethics approval and consent to participate:** This article is based on previously conducted studies and does not contain any studies with human participants or animals conducted by any of the authors.

**Consent for publication:** All authors meet the International Committee of Medical Journal Editors (ICMJE) criteria for authorship for this article, take responsibility for the integrity of the work as a whole, and have given their approval for this version to be published.

**Consent to participate:** Not applicable. This study was a systematic review and meta-analysis of previously published data and did not involve the recruitment of participants or the collection of individual-level identifiable information. Therefore, informed consent to participate was not required.

**Availability of data and materials:** Data analysis is available upon request.
