# Supplementary material for: Evaluation of the retinal and choroidal microvasculature in retinitis pigmentosa using OCT-angiography: a meta-analysis
Source: Ther Adv Ophthalmol. 2026 Sep 1;18:25158414261471361. doi: 10.1177/25158414261471361 (PMC13539038; doi:10.1177/25158414261471361)
Supplement: Supplementary file 1 [file sj-docx-1-oed-10.1177_25158414261471361.docx]

**Supplementary section**

Figure 1. Retinal capillary plexus perfusion in foveal area.

a. Funnel plot
b. Sunset power enhanced funnel plot

Figure 2. Retinal capillary plexus perfusion in parafoveal area.

a. Funnel plot
b. Sunset power enhanced funnel plot

Figure 3. Choriocapillaris perfusion in foveal and parafoveal area.

a. Funnel plot
b. sunset power-enhanced funnel plot

Figure 4. Foveal avascular zone in superficial and deep capillary plexus.

a. Funnel plot
b. sunset power-enhanced funnel plot
